# T23. DYNAMICS OF NEURONAL METABOLISM AFTER THE ACUTE ONSET OF PSYCHOSIS – A TWO YEARS FOLLOW-UP 1H/31P-MR-SPECTROSCOPY STUDY IN NEUROLEPTIC NAïVE UHR TRANSITION PATIENTS

**DOI:** 10.1093/schbul/sby016.299

**Published:** 2018-04-01

**Authors:** Stefan Smesny, Diana Berberich, Alexander Gussew, Kerstin Langbein, Mario Walther, Juergen Reichenbach

**Affiliations:** 1 University Hospital Jena; 2 University of Applied Sciences Jena

## Abstract

**Background:**

Glutamatergic dysfunction, deregulated mitochondrial metabolism and alterations of membrane phospholipids are considered as core pathology of psychosis, and have been studied in schizophrenic illness using magnetic resonance spectroscopy (MRS). Combining 1H- and 31P-MRS, this study investigates these aspects in Ultra-high risk (UHR-T) patients right after transition to psychosis (T0) and after a two years interval (T1) in a naturalistic longitudinal design, including treatment as usual by cognitive-behavioral therapy (CBT) and pharmacotherapy with second generation antipsychotics.

**Methods:**

We applied 3 T chemical shift imaging (3D 31P-MRS, 2D 1H-MRS) and hippocampal single-voxel 1H-MRS in 29 neuroleptic-naïve UHR-T patients and 27 healthy controls matched for age and gender. Glutamate (Glu) and N-acetyl-aspartate (NAA) reflect neuronal functioning, phosphocreatine (PCr), adenosine triphosphate (ATP) and NAA indicate mitochondrial function and energy metabolism, and phosphomono- and diester indicate the balance of phospholipid synthesis (PME) and -breakdown (PDE). Psychopathology was assessed using the CAARMS, BPRS-E and SCL-90-R. Generalized linear mixed models were used to examine case-control differences in metabolite changes over time, and associations with clinical improvement.

**Results:**

At T0, cross-sectional analysis revealed decreased NAA, Glu and PME levels in the left dorsolateral prefrontal cortex (DLPFC) and thalamus of UHR-T patients as well as higher PCr and lower PDE levels in the right hippocampus. (ii) Follow-up analysis (T1) showed in patients a significant increase of Glu in the bilateral DLPFC and the right thalamus, while a decrease of PCr was observed in the right hippocampus.

**Discussion:**

The observed metabolite pattern at T0 likely reflects a hypofunction of glutamatergic neurons and a disturbance of membrane phospholipid turnover in fronto-thalamo-hippocampal networks during the first acute onset phase of psychotic illness. The pattern of changes at T1 is suggestive for an improvement of neuronal functioning in these networks that is caused by therapy, and presumably underlies the observed clinical improvement in terms of negative symptoms and cognitive impairment.

